# The colonic mucosal virome in inflammatory bowel disease reveals Crassvirales depletion and disease-specific virome features

**DOI:** 10.1080/19490976.2025.2539450

**Published:** 2025-08-03

**Authors:** Austin Yan, James Butcher, David R. Mack, Alain Stintzi

**Affiliations:** a Department of Biochemistry, Microbiology, and Immunology, Faculty of Medicine, University of Ottawa, Ottawa, ON, Canada; b School of Pharmaceutical Sciences, Ottawa Institute of Systems Biology, Ottawa, ON, Canada; c Department of Pediatrics, Faculty of Medicine, University of Ottawa, Ottawa, ON, Canada; d Inflammatory Bowel Disease Centre and CHEO Research Institute, Children’s Hospital of Eastern Ontario, Ottawa, ON, Canada

**Keywords:** Virome, gut microbiome, inflammatory bowel disease, gut mucosa

## Abstract

The mucosal virome is increasingly recognized for its potential role in shaping intestinal health and disease. Building on previous findings, we analyzed the mucosal virome from 51 individuals, including newly diagnosed treatment naïve participants with ulcerative colitis (UC), Crohn’s disease (CD), and non-inflammatory bowel disease (non-IBD) controls, incorporating longitudinal sampling for a subset of the participants. Viromes were highly individualized, with no shared or core components across participants. Unlike fecal virome studies, we observed no significant associations between mucosal virome diversity and mucosal inflammation, disease subtype, or sampling site. However, there was positive correlation between virome and bacteriome diversity, particularly in CD, suggesting the presence of dynamic interactions that influence microbial community structure. *Crassvirales* was abundant in the mucosa layer and, consistent with prior studies, *Crassvirales* abundance was reduced in IBD, irrespective of inflammation status or IBD subtype. These findings highlight their potential as biomarkers of virome health. Our data also revealed the potential presence of altered bacteriome-virome interactions and longitudinal sampling revealed a persistent subset of viruses, potentially shaping disease progression and remission dynamics. Our study underscores the importance of distinguishing microbial community dynamics across IBD subtypes and highlights *Crassvirales* as key players in mucosal immunity.

## Introduction

Inflammatory bowel disease (IBD) is a chronic, relapsing illness at the interplay of host genetics, environmental factors, and the gut microbiota – the collection of bacteria, viruses, fungi, and archaea in our gastrointestinal tract.^1–3^ While most microbiome studies have focused on bacteria, gut viruses are increasingly being studied due to the rapid development of new virome-targeted bioinformatic tools and databases.^4–6^ Our gut virome is individualized and primarily composed of bacteriophages; recent studies have explored the temporal development of our virome,^7,8^ identified new highly prevalent intestinal viruses,^9,10^ studied the ecological stability of gut viruses,^11^ and investigated the virome’s role in human health.^12–14^

Changes to the gut virome have been reported in the IBD subtypes Crohn’s disease (CD) and ulcerative colitis (UC). These observations include the enrichment of temperate phages^15,16^ and reductions in the diversity of both DNA and RNA viromes in CD.^17^ *Crassvirales*, a viral order within the class *Caudoviricetes* (i.e. tailed bacteriophages) that are highly abundant in humans throughout life, are depleted in individuals with IBD.^11,18,19^ Viral communities with low diversity and a high abundance of non-*Crassvirales Caudoviricetes* have been linked to lower likelihood of achieving endoscopic remission, as well as an altered bacteriome.^20^ Other researchers, however, have suggested that high interpersonal variability in the virome may limit our ability to discern differences between the IBD and non-IBD virome.^21^

As with other microbiome analyses, a critical question remains: do observed correlations indicate causation? This question is particularly relevant in the context of intestinal mucosal inflammation as this is often considered a hallmark of IBD. Interestingly, clinical studies in participants with chronic-relapsing *C. difficile* infection have shown potential benefits when receiving virome-containing fecal filtrates (and thus devoid of other microbiome components) and virome alterations appear to be associated with fecal microbiome transplant responses.^22,23^ Furthermore, a recent study using a mouse model of colitis reported exacerbated inflammation in response to treatment with fecal virus-like particles derived from human participants with UC.^24^ Conversely, fecal virome transplantation from healthy human donors ameliorated colitis symptoms in a mouse model, whereas virome transplantation from IBD donors exacerbated inflammation.^25^ Together, these findings highlight the potential functional and causal role of the virome in modulating intestinal inflammation and underscore the need for further investigation into its contribution to IBD pathogenesis.

Virome analysis on rectal mucosa biopsies have demonstrated a decrease in *Caudovirales* diversity, richness, and evenness in UC participants.^16^ Studies that incorporate sampling throughout the colon are scarce as most IBD virome studies have limited their sampling to stool, but the few studies available support the hypothesis that the intestinal mucosa-associated virome is different from their luminal or fecal counterparts.^26^ Similarly, we have previously demonstrated that the colonic mucosal-luminal interface (MLI) fosters a unique viral population that included highly abundant *Crassvirales* phages in the proximal and distal colon that were not detectable in stool.^27^

Here, we expand our study of the MLI microbiome to evaluate the IBD colonic mucosal virome by using MLI samples collected from both the proximal and distal colon during diagnostic colonoscopy. We report significant alterations in the virome associated with IBD subtypes and different stages of disease activity. This approach provided a spatially resolved picture of the IBD virome that cannot be captured by fecal sampling alone.

We also assessed bacteriome-virome interactions in matched samples, identified a reduction in *Crassvirales* in IBD participants, and conducted a preliminary analysis of the MLI virome’s longitudinal stability. Our findings significantly enhance our understanding of the colonic mucosal virome in IBD, highlighting the utility of using MLI samples over stool samples to gain a more precise and comprehensive insight into the virome’s role in IBD pathology.

## Materials and methods

### Participants

A cross-sectional cohort of 51 pediatric participants were recruited between April 2018 and December 2019. This cohort comprised 35 children newly diagnosed with IBD [CD: *n* = 21 (60%); UC: *n* = 14 (40%)]. Additionally, 16 participants who underwent diagnostic colonoscopy for suspected IBD, based on presenting signs and symptoms, were included. These participants exhibited normal mucosal visual appearance during colonoscopy and showed no histological evidence of inflammation in mucosal biopsy specimens, thus meeting the criteria as a non-IBD control group. All participants were IBD treatment naïve at the time of their initial diagnostic colonoscopy and had mucosal luminal interface (MLI) aspirate sampling performed at both the proximal and distal colon during their procedure. Exclusion criteria for study recruitment and collection of MLI aspirates included (a) presence of diabetes mellitus, (b) presence of documented or suspected infectious diarrhea within the previous two months, and (c) use of antibiotics or probiotics within the past 4 weeks.

Diagnosis of CD or UC was made following thorough clinical, endoscopic, histologic, and radiologic evaluations according to standardized criteria to reduce observer bias of disease sub-type, location and severity.^28^ Disease location was described using the Montreal modification to the Paris classification system.^29^ For CD, the clinical severity at initial colonoscopy was reported using the Pediatric Crohn’s Disease Activity Index,^30^ and categorized as: mild (PCDAI = 11–30), moderate (PCDAI = 30–37.5), or severe (PCDAI ≥40). For UC, the Pediatric Ulcerative Colitis Activity Index^31^ was used to categorize disease severity as remission (PUCAI < 10), mild (PUCAI = 11–34), moderate (PUCAI = 35–64) or severe (PUCAI ≥65). Active colonic IBD (i.e., inflamed mucosa) was described by the visual appearance of colonic mucosa during colonoscopy (e.g. the loss of normal blood vessel appearance, mucosal erythema with mucosal ulcers) and was scored using the Simplified Endoscopic Score for Crohn’s disease (SES-CD)^32^ or the Modified Mayo Endoscopic Subscore for UC.^33^ Several participants (*n* = 8) required follow-up colonoscopy as part of their medical care, and regional MLI aspirates samples were also obtained during these serial colonoscopes. Follow-up CD clinical disease activity was based on use of the weighted PCDAI (wPCDAI).^34^ Clinical disease severity was categorized as remission (wPCDAI < 12.5), mild (wPCDAI = 12.5–39.5), moderate (wPCDAI = 40–57.5), or severe (wPCDAI > 57.5). Clinical data was managed using REDCap, hosted at the CHEO Research Institute. REDCap is a secure, web-based application designed to support data capture for research studies.^35^ Choice of therapy for patients was based on published guidelines^36,37^ to minimize variation in care with the final therapeutic choices and was determined collaboratively by the treating physician, patient, and family. Three participants (UC-F, UC-G, and UC-H) were previously described in Yan *et al*.^27^

### Ethics approval

Participants were prospectively enrolled, and samples were collected, as part of a larger Children’s Hospital of Eastern Ontario Review Ethics Board (REB) approved study (#09/37X). Informed written consent/assent were obtained from parents and/or participants, as appropriate.

### Sample processing and VLP extraction

Mucosal-luminal interface (MLI) aspirates were obtained during colonoscopy as previously described.^27,38,39^ In brief, mucosal-luminal interface (MLI) aspirates (40–80 mL) were collected from participants during their diagnostic endoscopy with sampling either being performed or supervised by a single physician to reduce sampling bias. Colonoscopy was performed following a one-day standard colon clean-out preparation,^40^ first aspirating and discarding any existing fluid and luminal debris. Sterile water was then flushed through the colonoscope onto the mucosa of the site of interest (i.e. PC or DC) and aspirated through the colonoscope into a sterile container. These regional MLI samples were collected from both the PC and DC of each participant, immediately placed on ice in the endoscopy room, transported to the laboratory, and aliquoted within 30 minutes for storage at −80°C until further processing.

Aliquots of 10 mL were used for virus-like particle (VLP) purification, which involved the following steps: centrifugation and sequential filtration with 5.0 and 0.45 µm filters (Sigma-Aldrich, SLSV025LS and SLHV033RB) to remove cells and debris; virus-like particle precipitation with 10% w/v PEG-8000 (Fisher Scientific, BP233); resuspension in saline-magnesium buffer and bacterial cell lysis with 1 mg/mL lysozyme (Sigma, L4919), then chloroform treatment; centrifugation and DNase and RNaseI (Thermo Scientific, AM2238, Life Technologies, EN0602) treatment of the supernatant to degrade remaining bacterial nucleic acids; and VLP lysis with Proteinase K and cetyltrimethylammonium bromide (Fisher Scientific, BP1700 and O3042) and viral DNA extraction with phenol-chloroform-isoamyl alcohol (25:24:1, pH 6.7).

Viral nucleic acids were purified from the aqueous layer using the Dneasy Blood and Tissue Kit (QIAGEN 69,506), eluted in 50 µL of water, and subjected to centrifugal vacuum concentration to maximize input DNA for the multi-displacement amplification (GenomiPhi V2: GE Life Science 25,660,032). Reactions using 1 µL of input DNA were performed in triplicate, pooled, and purified using the Dneasy Blood and Tissue Kit. DNA quantification was performed using the Qubit dsDNA HS Assay Kit (Thermo Fisher, Q32854).

### Virome sequencing and host-read removal

Shotgun DNA sequencing was performed at the Génome Québec CES using the NEB Ultra II library preparation kit and Illumina NovaSeq 6000 platform as previously described.^27^ In summary, raw sequencing reads were trimmed and filtered using Cutadapt 2.10 (Illumina’s universal adapters: AGATCGGAAGAG; AGATCGGAAGAG) and Trimmomatic 0.36 (SLIDINGWINDOW:4:20, MINLEN:60, and HEADCROP:10). Reads mapping to the human genome with bowtie2 (GRCh38 using ultra-sensitive mode)^41^ were removed, along with low complexity reads using komplexity.^42^ Bacterial contamination was assessed by aligning reads to the *cpn60* database with bowtie2’s ultra-sensitive mode.^43^

### Metaviromic analysis

Host-decontaminated, high-quality reads from each virome sample were assembled using MEGAHIT v1.2.7^44^ with a minimum contig length of 3 kb. These reads were then mapped to the assemblies using bowtie2. The normalized relative abundance (NRA) of each viral contig was calculated as $\frac{x_{i}}{\underset{\;}{\sum }x_{i}}$ where x_i_ is the number of reads mapping to a contig divided by the contig length.

We used a similar approach to Yutin *et* *al.*^45^ to identify *Crassvirales* contigs, specifically by selecting contigs harboring all three universally conserved *Crassvirales* genes: portal protein, large terminase subunit (TerL), and major capsid protein (MCP). Open reading frames were predicted using Prodigal v2.6.3^46^ in metagenome mode, with subsequent clustering using vContact2.^47^ Further functional annotation of viral open reading frames (ORFs) was performed with Prokka,^48^ with mapping to predicted ORFs to VOG (version 211), pVOG, PFAM, TIGRFAM, Kegg, and crAssphage databases.^45^ Viral contigs were also identified using geNomad 1.5.0 with score-calibration enabled^49^ and evaluated using CheckV.^50^

### 16S rRNA amplicon sequencing and analysis

DNA extraction, V6-16S rRNA gene library construction and sequencing were performed as previously described.^38,51^ In short, 2 mL of MLI sample was pelleted and metagenomic DNA extracted using a Fast-DNA Spin Kit. Extracted DNA was normalized to 5 ng/μL and stored at −20°C until library construction. V6-16S library construction was carried out using two successive rounds of PCR, amplicons purified using a MilliPore™ MultiScreen™ PCR96 plate and samples pooled at equimolar ratios. Pooled libraries were gel purified using a QIAquick PCR Purification Kit and then sequenced on an Illumina HiSeq 2500 with 100 bp paired-end reads at The Center for Applied Genomics (Toronto, Canada). Paired-end sequencing reads were demultiplexed and PCR primers removed using cutadapt with paired-reads that contained an ambiguous base-call or were < 50 bp in length discarded. Subsequent analysis was performed in R using dada2. Paired-reads were quality filtered to remove pairs with > 1 expected error and those aligning to the phiX genome. Forward and reverse reads were denoised separately for each sequencing run. Denoised reads were merged, the merged reads from each run combined together and chimeras identified using pooling on the entire dataset to generate amplicon sequence variants (ASVs). ASVs were subsequently analyzed using phyloseq.

### Statistical analysis

Alpha-diversity and beta-diversity analysis, statistical analysis, and plotting were performed in R 4.1.3 using phyloseq 1.36.0,^52^ reshape2 1.4.4,^53^ ggplot2 3.3.0,^54^ ggthemes 4.2.0,^55^ and ggpubr.^56^ Alpha diversity analysis was performed using read counts rarefied to 11,390,062 reads per sample for metavirome analysis (the lowest number of viral reads identified in a sample across the dataset) and 13,915 sequences for ASV analysis. DESeq2 was used to identify differentially abundant ASVs between the non-IBD controls and IBD-subtypes with biological sex (male/female) and sampling site (PC/DC) used as covariates in the analysis (Supplementary Table S1), fold changes ≥ 2 with adjusted *p* values < 0.05 were considered significant. To maximize our potential to identify low abundant ASVs with differential abundance, we kept ASVs present at > 2 reads in at least 4% of the samples and rarefied the dataset to 100,000 reads, removing samples below this threshold. Viral beta-diversity analysis was performed at the viral cluster level (using vContact2 gene-sharing networks) and at the ASV level for the bacteriome. Differential abundance analysis of viral taxa annotations using geNomad was performed with the Wilcoxon test or Kruskal-Wallis test with false-discovery rate correction; viral taxa present in fewer than three samples were excluded from this analysis. Spearman correlations between bacterial genera and viral order/classes were performed at diagnosis. PC/DC samples were merged with only those containing detectable reads for both the bacterial genus and viral order/class considered. Bacterial-viral pairs with ≤ 3 positive samples were omitted from the analysis. Correlations were done using all participants and each participant group separately (non-IBD, CD, UC).

## Results

### Study population

There were no statistical differences in age at diagnostic colonoscopy (i.e., diagnosis) between the three groups of participants (CD, UC, and non-IBD) or between the number of males and females in each group (Table 1). Individuals without IBD (non-IBD) were participants undergoing colonoscopy to assess for IBD based on signs and symptoms but ultimately found to have normal mucosal appearance and normal mucosal biopsy histology. There was a similar number of participants with an inflamed proximal colon (PC) in either CD or UC (39% vs. 31%) and as expected there was more UC participants with an inflamed distal colon (DC) than in CD participants (90% vs. 47%). There was one participant with UC that did not have DC inflammation and was ultimately diagnosed with proctitis. This participant thus had their DC sampling conducted proximal to the site of inflammation. Longitudinal regional colonic MLI samples were available for some participants (*n* = 8, Table 2) as these individuals required a repeat colonoscopy as part of their clinical care.

**Table 1.** Participant and sample summary at time of initial diagnostic colonoscopy. sd: standard deviation; PC: proximal colon; DC: distal colon; CD: Crohn’s disease; UC: ulcerative colitis; L1: distal 1/3 ileal ± limited cecal disease; L2: colonic; L3: ileocolonic; E1: ulcerative proctitis; E2: left-sided UC; E3: extensive UC; E4: pancolitis.

	Non-IBD	CD	UC	*p value*
Participants (n)	16	21	14	NA
Age (s.d.)	13.4 (4.2)	14.0 (2.6)	12.9 (3.5)	*p* = 0.74
Sex (% female)	7 (44%)	14 (66%)	6 (43%)	*p* = 0.27
Disease activity (based on PCDAI or PUCAI)	NA	Mild: 2 Moderate: 9 Severe: 3	Mild: 2 Moderate: 9 Severe: 3	NA
Inflammation location	NA	L1: 9 L2: 5 L3: 7	E1: 1 E2: 3 E3: 7 E4: 3	
Number of samples per site:				
PC (n, % inflamed)	16 (0, 0%)	18 (7, 39%)	13 (4, 31%)	*p* = 0.02 (for inflamed between CD & UC)
DC (n, % inflamed)	14 (0, 0%)	17 (8, 47%)	10 (9, 90%)	*p* = 0.00006 (for inflamed between CD & UC)

**Table 2.** Longitudinal sample collection of the mucosal-luminal interface virome. Collection time reflects the number of weeks from initial diagnostic endoscopy. 5-ASA: mesalamine, 6-MP: mercaptopurine, AZA: azathioprine, MTX: methotrexate, PRED: prednisone, IFX: infliximab.

Diagnosis and participant	Collection time (weeks)	Clinical severity score	Site	Local score (SES-CD or MAYO-UC)	Treatment (induction/maintenance)
CD-A	0	PCDAI 57.5 (severe)	PC	0	-
			DC	0	
	17	wPCDAI 10 (remission)	PC	0	PRED/AZA +6-MP
			DC	0	
CD-E	0	PCDAI 45 (severe)	PC	**0**	-
	21	wPCDAI 57.5 (moderate)	PC	**1**	PRED/AZA +6-MP
CD-H	0	PCDAI 30 (moderate)	PC	3	-
			DC	6	
	14	wPCDAI 0 (remission)	PC	0	PRED/AZA +6-MP
CD-I	0	PCDAI 35 (moderate)	PC	0	-
			DC	0	
	21	wPCDAI 20 (moderate)	PC	0	PRED/MTX
			DC	0	
CD-M	0	PCDAI 32.5 (moderate)	PC	0	-
	33	wPCDAI 42.5 (moderate)	PC	0	PRED/MTX
CD-Q	0	PCDAI 60 (severe)	PC	5	-
	19	wPCDAI 35 (mild)	PC	0	IFX/IFX
UC-A	0	PUCAI: 35 (Moderate)	PC	Grade 1	-
	24	PUCAI: 15 (Mild)	PC	Grade 0	
			DC	Grade 2	
UC-G	0	PUCAI: 30 (Mild)	PC	Grade 0	-
			DC	Grade 1	
	17	PUCAI: 40 (Moderate)	PC	Grade 0	PRED/5-ASA
			DC	Grade 2	
	37	PUCAI: 40 (Moderate)	DC	Grade 2	PRED/5-ASA
	63	PUCAI: 0 (Remission)	PC	Grade 0	IFX/IFX
			DC	Grade 0	

### Isolation of colonic viromes

Virome sequencing was performed on 88 MLI samples from the PC and/or DC from 51 individuals at time of initial diagnostic colonoscopy. We identified 87,658 viral contigs (VCs), which were subsequently annotated and assessed for quality. Among these, a subset of 4,793 VCs (5.5% of all VCs) were classified as “complete” or “high-quality” (both hereafter referred to as “high-quality”). These high-quality VCs accounted for the majority of mapped reads (mean of 69.5% for new-onset samples), consistent with previous findings.^27^

**Figure 1.**
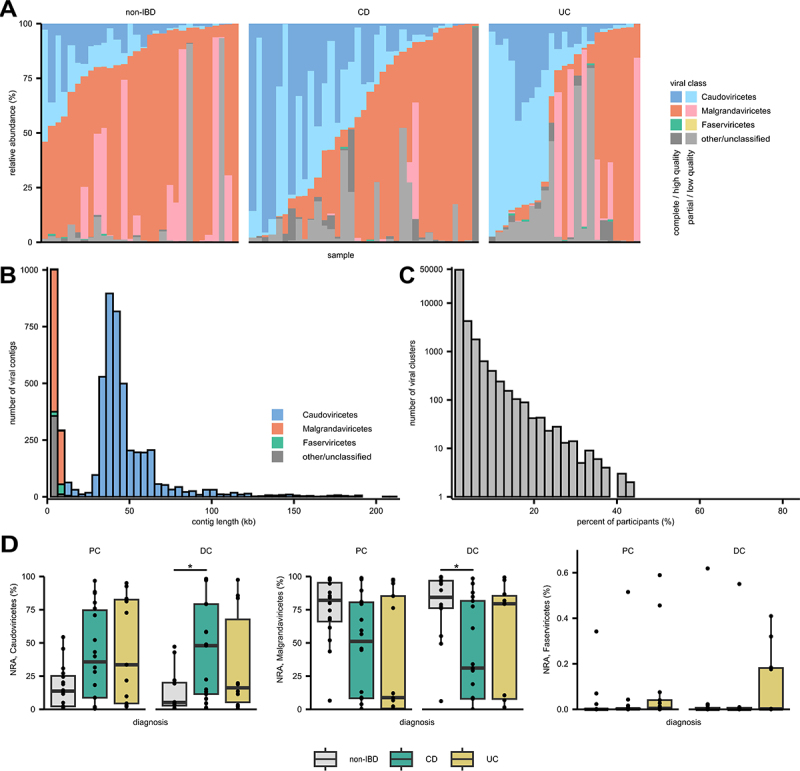
Taxonomic annotation and clustering of viral contigs. Virome sequencing of study participants undergoing their initial diagnostic colonoscopy revealed 87,658 viral contigs which were subject to taxonomic annotation and gene-network clustering. (A) normalized relative abundance of viral contigs per sample, grouped by diagnosis. (B) histogram showing contig length of high-quality VCs based on viral class. (C) histogram showing the percent of participants that share each viral cluster. (D) normalized relative abundance of *Caudoviricetes*, *Malgrandaviricetes*, and *Faserviricetes* by site and diagnosis. CD: Crohn’s disease; UC: ulcerative colitis; PC: proximal colon; DC: distal colon; *: *p* < 0.05 (pairwise comparisons with false-discovery rate performed for each site and viral class); comparison bar not shown if not significant.

Viral contigs were annotated using geNomad, which reflects recent changes to viral taxonomy nomenclature.^49,57^ The majority of annotated VCs belonged to the class *Caudoviricetes*, comprising 31,798 VCs, of which 3,346 were high-quality VCs (Figure 1A). This class previously included the order *Caudovirales* and its morphological subfamilies *Siphoviridae*, *Myoviridae*, and *Podoviridae* (these taxa are now abolished), and now includes the new order *Crassvirales*.^57^ The second most common viral class was *Malgrandaviricetes*, with 894 VCs, of which 784 were high-quality VCs (Figure 1A). Nearly all of these were identified as *Microviridae* (892 of 894 VCs).

*Caudoviricetes* and *Malgrandaviricetes* were the most abundant and prevalent viral classes, present in 87/88 (98.9%) and all 88 specimens, respectively (Figure 1A). Other than those two aforementioned classes, only *Faserviricetes* was detected in more than 25% of samples with 50/88 (56.8%) albeit at low abundance (mean 0.05% relative abundance; maximum 0.62% relative abundance). The remaining VCs were predominantly ssDNA viruses from the viral families *Circoviridae* (class *Arfiviricetes*), *Inoviridae* (class *Faserviricetes*), and *Genomoviridae* (class *Repensiviricetes*). Of the high-quality genomes, most ssDNA viruses, including *Microviridae*, were 3–5 kb in size, whereas most *Caudoviricetes* ranged from 10–100 kb (Figure 1B).

The 87,658 VCs were further categorized into 56,515 viral clusters (VCLs) using a gene-network approach to facilitate interparticipant comparison.^47^ Most viral clusters (*n* = 48,674, 86.1%) were only present in one participant (Figure 1C). Very few VCLs were present in > 10% of all participants (*n* = 786, 1.39%), and only five VCLs (0.01%) were present in > 50% of all participants.

### Alterations in the treatment-naïve colonic virome in inflammatory bowel disease

**Figure 2.**
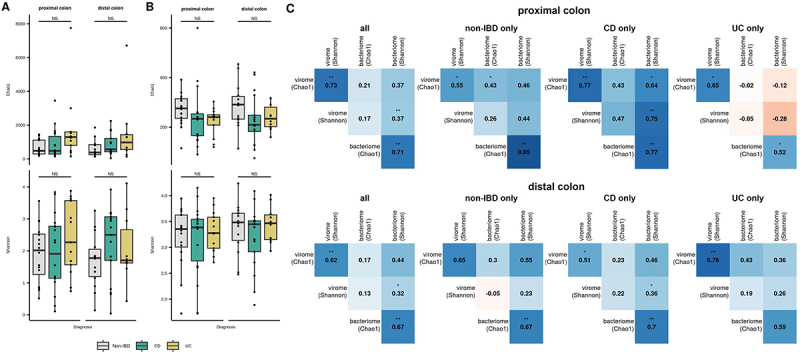
Alpha-diversity of the mucosal-luminal interface virome and bacteriome in inflammatory bowel disease. Chao1 index and Shannon diversity were calculated based on rarefied counts to (A) viral contigs and (B) 16S rRNA amplicon sequence variants. (C) correlations between bacterial vs viral alpha diversity at the proximal and distal colon, including all samples and separated by disease status. **, *p* < 0.01; *, *p* < 0.05; NS: not significant; CD: Crohn’s disease; UC: ulcerative colitis.

Within the two main viral classes, *Caudoviricetes* and *Malgrandaviricetes*, we observed a trend for increased *Caudoviricetes* among participants with IBD (Figure 1A,D), though this was only statistically significant between CD and non-IBD participants at the DC (Figure 1D). Conversely, *Malgrandaviricetes* were significantly enriched in DC samples from non-IBD participants compared to participants with CD (Figure 1D). Analysis of geNomad viral order/family annotations revealed no significant differences in viral taxa between UC, CD, and non-IBD participants or by local inflammation (Supplementary Table S2). We also evaluated the viral and bacterial community diversity using the Chao1 and Shannon diversity indices, which reflect species richness and evenness, respectively. No significant differences were observed in Chao1 or Shannon diversity among viral or bacterial communities based on diagnosis or inflamed mucosa (Figure 2A,B, Supplementary Figure S1). Although there was a trend toward decreased viral richness and diversity in samples obtained from inflamed mucosa and in participants with CD with a higher clinical activity based on PCDAI score, these results were not statistically significant (Supplementary Figure S1). Additionally, no statistically significant differences were identified when viral communities were partitioned into *Caudoviricetes* and non-*Crassvirales Caudoviricetes* groups. There was a positive correlation between the Shannon diversity of the virome and bacteriome in both the PC (Spearman correlation 0.37, *p* = 0.004) and DC (Spearman correlation 0.32, *p* = 0.013). When subsetted by IBD subtype, this correlation was higher and statistically significant in participants with CD but was reduced in participants with UC, even trending toward an inverse relationship at the PC (Figure 2C).

**Figure 3.**
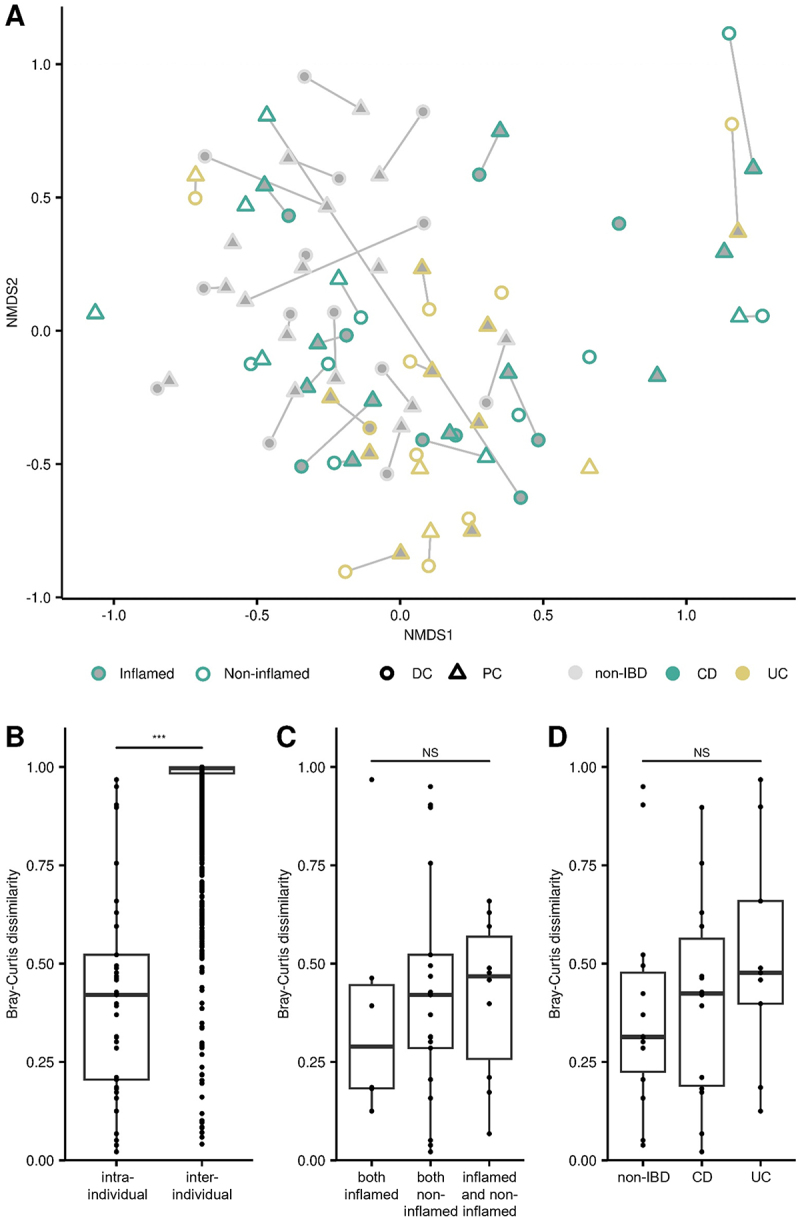
Virome beta-diversity at the mucosal-luminal interface in inflammatory bowel disease. (A) virome community structure visualized with NMDS plot (stress = 0.251, k = 2) with annotations for site, diagnosis, and local inflammation. (B) Bray-Curtis dissimilarities were calculated and compared between and within individuals. Dissimilarities between proximal and distal colon sites from the same participant were compared based on (C) local inflammation and (D) diagnosis. ***, *p* < 0.001; NS: not significant; CD: Crohn’s disease; UC: ulcerative colitis.

**Figure 4.**
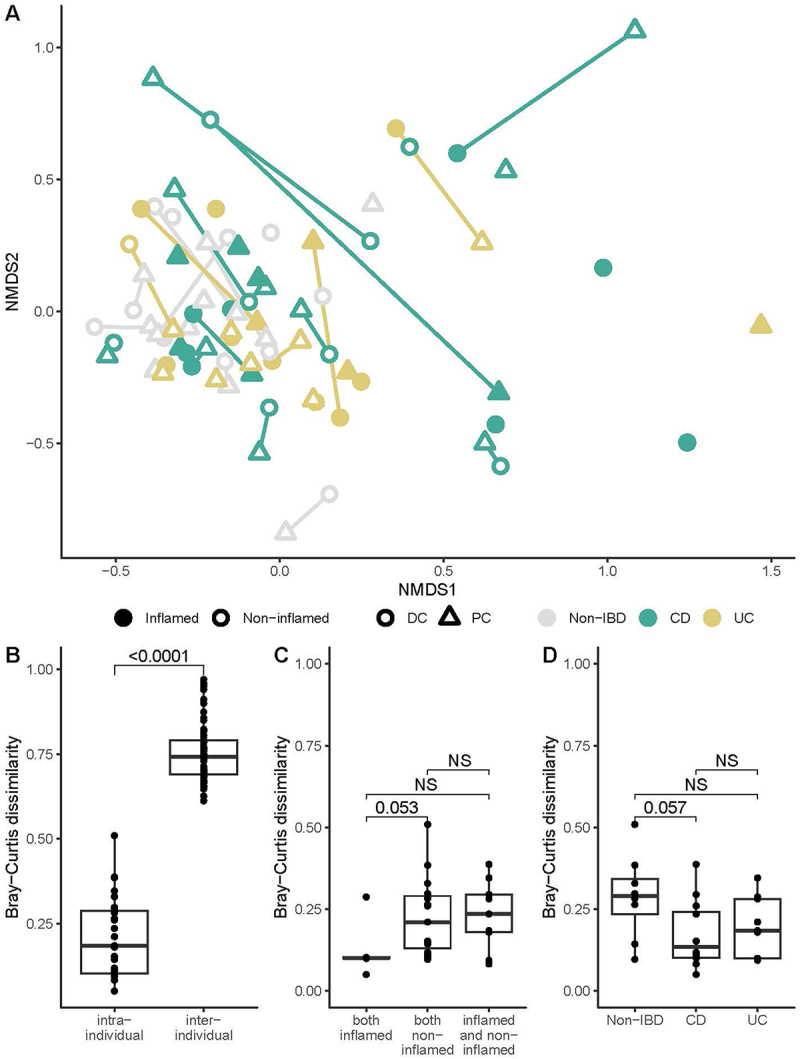
Bacteriome beta-diversity at the mucosal-luminal interface in inflammatory bowel disease. (A) bacteriome community structure visualized with NMDS plot (stress = 0.193, k = 2) with annotations for site, diagnosis, and local inflammation. (B) Bray-Curtis dissimilarities were calculated and compared between and within individuals. Dissimilarities between proximal and distal colon sites from the same participant were compared based on (C) local inflammation and (D) diagnosis. ***, *p* < 0.001; NS: not significant; CD: Crohn’s disease; UC: ulcerative colitis.

Beta-diversity analysis did not reveal distinct viromes based on the colonic site of MLI collection, mucosal inflammation, or IBD subtype (Figure 3A, Supplementary Table S3). Instead, our data indicates that mucosal viromes are highly individualized, with significantly reduced intra-individual Bray-Curtis dissimilarity (i.e. between one’s PC and DC) compared to inter-individual comparisons (*p* < 2E–16, Figure 3B). There was no significant difference for intra-individual comparisons based on local inflammation (Figure 3C) or IBD subtype (Figure 3D). When analyzing the bacteriome data using the same methods, we found that there is a greater trend for participants with IBD to cluster away from non-IBD controls (Figure 4A, Supplementary Table S3); although this explained a minimal amount of the overall variation (R^2^ = 0.04). We also found that participant bacteriomes were personalized (Figure 4B), although not to the same extent as seen for the virome (median inter-individual Bray Curtis dissimilarity of 0.75 versus almost 1.0 for the virome). There was also no difference between the bacteriome intra-individual comparisons based on local inflammation (Figure 4C) or IBD subtype (Figure 4D).

### High abundance of *Crassvirales* in the colonic virome depleted in the proximal colon in IBD

**Figure 5.**
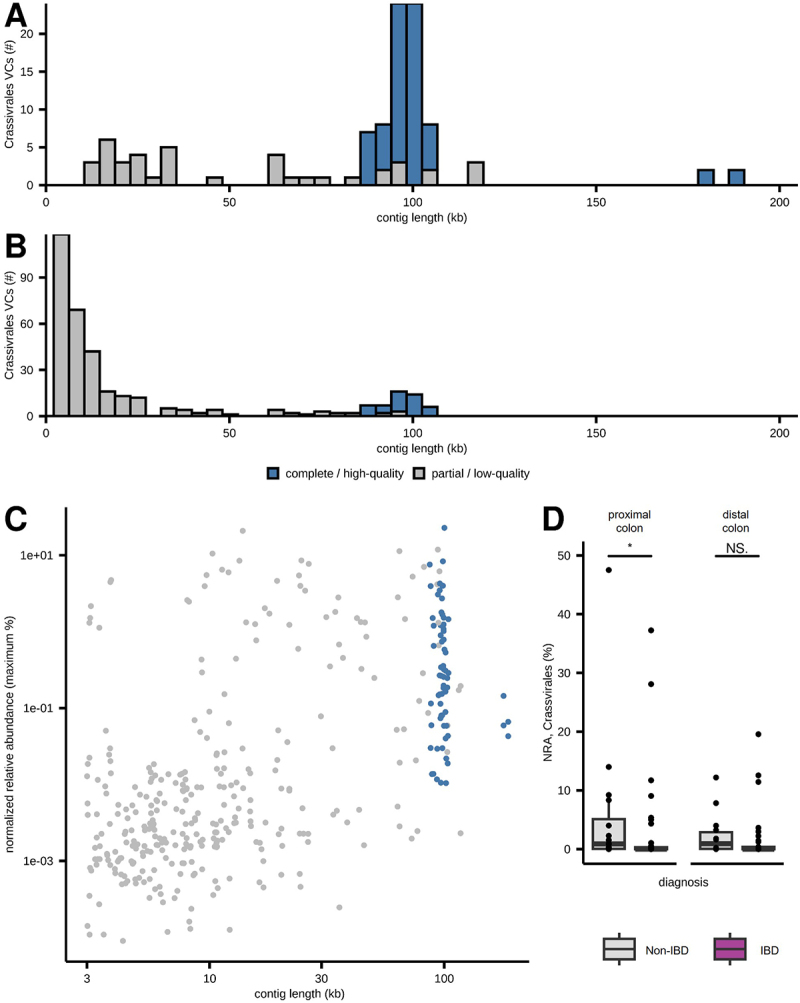
*Crassvirales* at the human mucosal-luminal interface. (A) quality and length of *Crassvirales* VCs identified using presence of portal protein, TerL, and MCP. (B) *Crassvirales* VCs annotated using geNomad. (C) relative abundance vs. length of *Crassvirales* VCs at the human MLI. (D) normalized relative abundance of *Crassvirales* at the proximal and distal colon MLI in participants with and without IBD.

*Crassvirales* are a viral taxon of high interest among recent virome studies. By using a similar approach to Yutin *et* *al.*^45^ we identified 109 *Crassvirales* VCs in samples collected during the initial diagnostic colonoscopy (Figure 5A). Most of these were high-quality VCs (68/109) and range in size from 90 to 105 kb (64/109). Taxonomic annotations by geNomad^49^ identified a greater number of *Crassvirales* VCs (*n* = 350). However, these primarily included shorter and lower quality contigs: only 45/350 were complete or high-quality and only 43/350 were greater than 90 kb (Figure 5B). Combining the two methods yielded a set of 386 *Crassvirales* VCs (shown in Figure 5C) which were used to evaluate the relative abundance of *Crassvirales*.

*Crassvirales* were identified in 40/51 study participants, including 15/21 participants with CD, 11/14 participants with UC, and 14/16 participants without IBD. Interestingly, the normalized relative abundance of *Crassvirales* was significantly higher in the PC of participants without IBD than in participants with IBD, with the DC showing a similar, but not statistically significant, trend (Figure 5D). There was no significant difference in *Crassvirales* levels between participants with UC and CD or between the PC and DC in this dataset. This significant difference was also not observed when only geNomad annotations were used (Supplementary Table S2).

### Alterations in the colonic bacteriome

**Figure 6.**
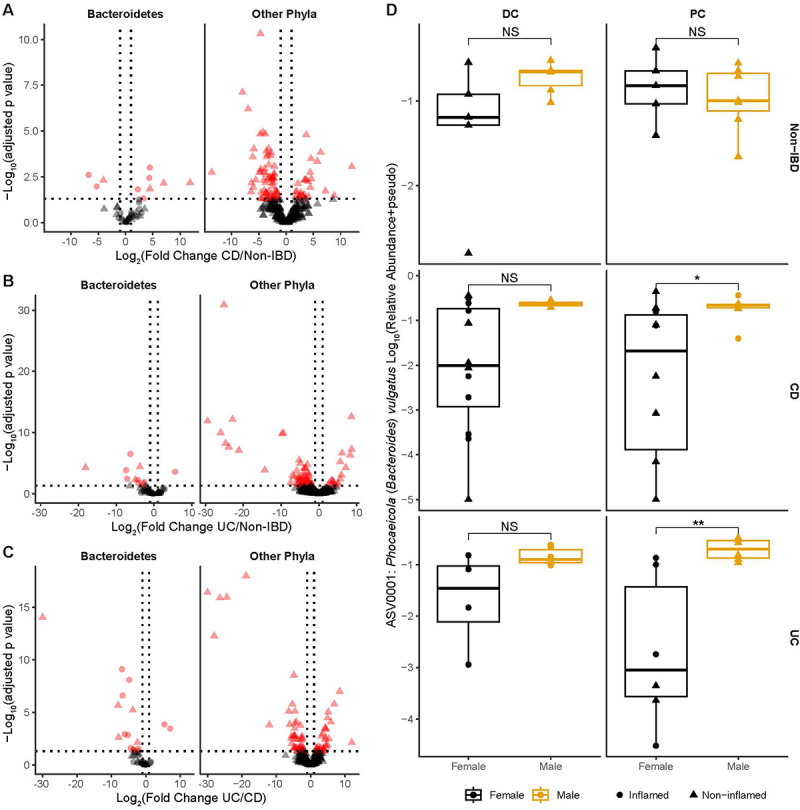
Differentially abundant ASVs between non-IBD controls and IBD subtypes. ASVs identified by DESeq2 as differentially abundant (absolute Log_2_ Fold change ≥ 1, adjusted *p*-value ≤0.05) for comparisons between CD and non-IBD controls (A), UC and non-IBD controls (B), and UC and CD (C). ASVs belonging to Bacteroidetes are displayed separately from those of other phyla. Relative abundances of ASV0001 (putative *Phocaeicola vulgatus*) are shown for male and female participants in non-IBD, CD, and UC groups across sampled sites. Statistical differences between biological sexes were assessed using the Mann-Whitney U test * *p* < 0.05; ** *p* < 0.01; NS: not significant.

As we observed potential alterations in *Crassvirales*, we were interested in whether there were corresponding alterations in the colonic bacteriome of these specific participants that followed the same trends. We identified 108, 96 and 99 amplicon sequence variants (ASVs) differentially abundant between CD vs non-IBD, UC vs non-IBD and UC vs CD after controlling for sex and colonic sampling region (Supplementary Table S1). There were more ASVs belonging to the phylum Bacteroidetes, and in particular those belonging to the genera *Bacteroides/Phocaeicola* that were enriched in participants with CD as compared to either UC or non-IBD (Figure 6A–C). In contrast, participants with UC had more Bacteroidetes ASVs depleted as compared to either non-IBD or CD. Interestingly, the dominant Bacteroidetes ASV matched with 100% identity across its entire length to *Phocaeicola* (formally *Bacteroides) vulgatus* and was enriched in both CD and UC subtypes as compared to individuals without IBD. This ASV was also enriched in participants with CD as compared to those with UC. Moreover, we noted striking sex differences in the abundance of this ASV regardless of IBD sub-type (Figure 6D), with males having higher levels as compared to their female counterparts (although this only reached significance at the PC). In contrast, there was no difference seen between the sexes in those without IBD. We also identified an ASV which matched perfectly to *Phocaeicola* (formally *Bacteroides) dorei* in our dataset (ASV0002), but this ASV was not found to be differentially abundant. We also investigated potential correlations between ASVs and genera and *Crassvirales* abundances across different participant groups and sampling sites. However, no statistically significant correlations were detected in any group or site. To further identify potential bacteriome-virome correlations, we preformed a high-level analysis by assessing whether there were specific bacterial genera that correlated with viral clusters at the Order/Class levels. These analyses revealed numerous potential genera-viral correlations (Supplementary Table S4), including several that appeared to have distinct patterns between non-IBD, CD or UC participants (Supplementary Figure S2). In particular, *Eggerthella* and *Faserviricetes* showed positive correlations in CD and negative correlations in UC while *Faecalibacterium* and *Caudoviricetes* were positively correlated in non-IBD and negatively correlated in CD and UC.

### Exploratory analysis assessing the temporal stability of the MLI virome

Longitudinal samples were available from eight IBD participants (6 CD, 2 UC), collected during repeat colonoscopies (Table 2). These samples were collected at a mean of 20.8 weeks after their initial diagnostic colonoscopy (ranging from 14.4–32.7 weeks). Participant UC-G had two additional samples obtained at 37 and 63 weeks post-diagnosis. The initial samples represented treatment-naïve viromes, while subsequent samples were influenced by varying illness severities and the introduction of different IBD treatments (i.e., corticosteroids, mesalamine, immunomodulators, and anti-TNFα monoclonal antibodies; Table 2). Thus, gene-sharing based analysis was used to cluster viral contigs for longitudinal comparisons.

**Figure 7.**
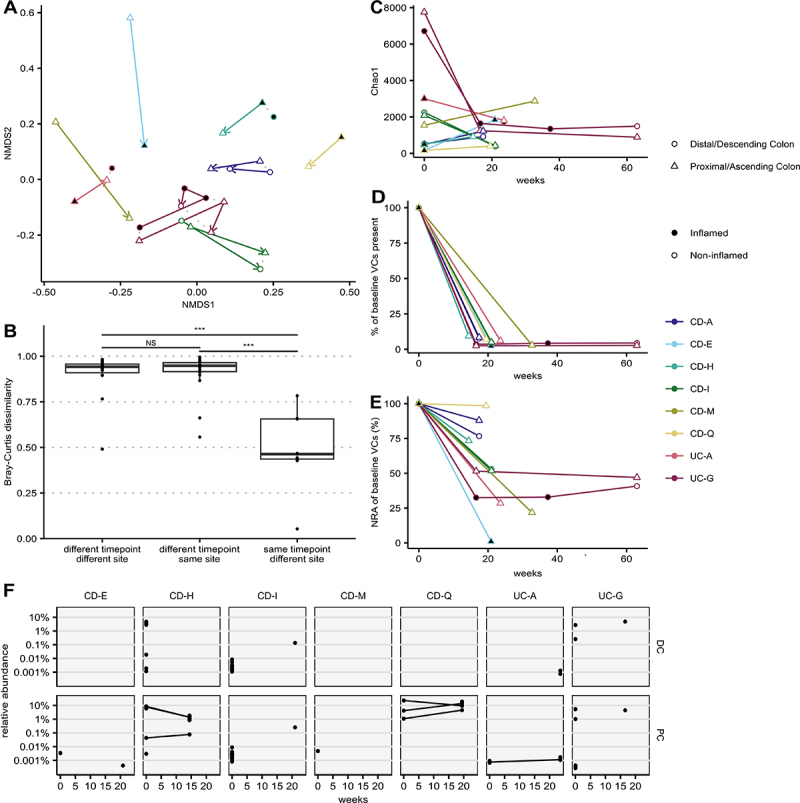
Longitudinal virome sampling at the MLI in participants with inflammatory bowel disease. (A) longitudinal virome community structure visualized by NMDS plot (stress = 0.175, k = 2) with annotations for participant, site, and local inflammation. Solid coloured lines represent samples from the same participant and site with arrows originating from the first sample; samples taken at the same timepoint are connected with a gray dotted line (B) intraparticipant Bray-Curtis dissimilarities compared between sites and time. NS: not significant; ***: *p* < 0.001. (C) Chao1 index of longitudinal virome samples. (D) percent of a sample’s viral clusters shared with the participant’s initial baseline sample at time of diagnostic endoscopy. (E) total normalized relative abundance of baseline viral contigs present. (F) normalized relative abundance of *Crassvirales* clusters identified by longitudinal virome sampling; only participant sites with multiple timepoints are plotted. Clusters representing same *Crassvirales* contigs are connected with lines. *p* < 0.001; PC: proximal colon, CD: Crohn’s disease; UC: ulcerative colitis.

This exploratory analysis revealed that intra-individual serial variations in viral communities exhibited significantly greater similarity compared to inter-individual variations (Figure 7A,B *p* < 2e-16). Within individuals, samples collected at different time points showed greater variation in viral community composition than samples collected simultaneously from different anatomical sites (e.g., proximal colon [PC] and distal colon [DC]; Figure 7B, *p* < 0.001). Furthermore, the overall viral diversity remained relatively stable over time within individuals when accounting for clinical disease severity (Figure 7C).

Between 2.4 and 9.3% of VCs identified at the initial diagnostic colonoscopy were detected at subsequent time-points (Figure 7D). These persistent VCs were more likely to be highly abundant, representing over 25% of the normalized relative abundance of VCs in most participants (Figure 7E). Two participants with serial MLI sampling showed initial VCs persisting at similar levels over the subsequent year.

*Crassvirales* VCs were detected in the colonic virome of 7/8 individuals with repeat samples available (Figure 7F). While most *Crassvirales* were identified at only one timepoint, several participants had *Crassvirales* VCs that were conserved longitudinally.

## Discussion

### Virome diversity at the mucosal-luminal interface

We previously demonstrated that the MLI virome is distinct from the stool virome.^27^ In this study, we build upon our previous work by expanding our cohort from three participants with UC to fifty-one individuals, including participants with UC and CD, and individuals without IBD. Furthermore, we performed longitudinal sampling for eight of these participants. The viromes within our cohort were highly personalized, with no shared or core virome identified across individuals.

A fecal virome study reported reduced overall viral alpha-diversity and increased diversity of *Caudovirales* (now classified under *Caudoviricetes*) in CD,^15^ although a re-analysis of the same dataset found no significant changes in overall viral alpha-diversity.^58^ In contrast, our dataset did not identify statistically significant associations between viral diversity and the presence of mucosal inflammation, CD or UC, or sampling site (i.e. PC vs. DC), even with targeted analysis of *Caudoviricetes* populations. These results are consistent with recent analysis of unamplified fecal viromes by Stockdale *et* *al.*,^21^ which suggested that high interpersonal variability may substantially confound alpha and beta-diversity comparisons in IBD virome studies, to a much greater extent than for bacteriome analyses. We did demonstrate an enrichment of *Caudoviricetes* in the distal colon of individuals with CD compared to individuals without IBD, with a corresponding decrease of *Malgrandaviricetes*. Viral taxa correlations with IBD subtype appeared to be stronger than correlations with local inflammation, suggesting that shifts in the mucosal virome may be more reflective of overall host disease than site-specific processes.

While Norman *et* *al.*^15^ initially reported an inverse relationship between bacterial and viral alpha-diversity, more recent fecal studies have shown a positive correlation between gut virome diversity and the bacteriome, an effect that is driven by bacteriophages rather than eukaryotic viruses.^59^ Our data also supports a positive correlation between virome and bacteriome alpha diversity, which appears to be primarily driven by our cohort of individuals with CD. This positive correlation suggests that a more diverse bacteriome may provide a broader ecological niche for bacteriophages to proliferate, potentially facilitating dynamic interactions between phages and their bacterial hosts. These interactions could promote bacterial turnover through lysis, thereby shaping microbial community composition and maintaining balance within the gut ecosystem. Alternatively, the increased diversity of bacteriophages may help stabilize bacterial communities by preventing the overgrowth of specific taxa via predator-prey dynamics. Conversely, our results in UC participants are consistent with those of Zuo *et* *al.*,^16^ who reported a weakening of bacterial-viral correlations in mucosal biopsies in individuals with UC compared to those without IBD.

In addition, we identified evidence for numerous bacteriome–virome interactions within the MLI, with several of these correlations displaying different directions depending on whether the participant was non-IBD, CD or UC (Supplementary Figure S2, Supplementary Table S4). As positive correlations typically reflect stable host – phage interactions and negative correlations suggest predatory – prey relationships, the reversal of these correlations in IBD suggest that normal bacterial-viral interactions have been disrupted.^60^ For example, *Eggerthella* and *Faserviricetes* showed opposite correlations in CD (positive) and UC (negative) with no correlation in non-IBD participants. To the best of our knowledge, there have not been any reports of direct *Faserviricetes-Eggerthella* interactions, however increased levels of *Eggerthella* have been reported in IBD cohorts and this genus has been reported to drive colitis via Th17 activation.^61–63^ We also identified *Faecalibacterium* as negatively correlated with *Caudoviricetes* in CD/UC and positively correlated in non-IBD. *Faecalibacterium* has been reported as being decreased in people with IBD, although we have found increases in *Faecalibacterium* at the MLI.^38,62,63^ Interestingly, the primary *Faecalibacterium* phages that have been reported thus far in the literature also belong to *Caudoviricetes*, suggesting that these findings may represent a direct bacterial–viral interaction.^64,65^ These disease-specific findings highlight the importance of recognizing the presence of distinct microbial pathogenesis and community structures in CD and UC. This disruption of bacterial–viral interactions may reflect alterations in the stability and functionality of the mucosal microbiota, potentially contributing to the disrupted microbial ecosystem. Altogether, these findings underscore the importance of distinguishing microbial community dynamics and pathogenesis across different IBD subtypes.

### *Crassvirales* at the mucosal-luminal interface

We used two distinct approaches to identify *Crassvirales* VCs. The VCs identified by geNomad represent short partial *Crassvirales* genomes that do not contain any of the three marker genes used by the first approach (Yutin *et* *al.*^45^) Despite these differences, these two approaches showed strong concordance. Among the 109 VCs containing the three universal *Crassvirales* genes, all were classified by geNomad within the class *Caudoviricetes*, with 73 further assigned to the order *Crassvirales*.

In our previous study, we identified two abundant *Crassvirales* species at the MLI that were nearly undetectable in matched stool samples,^27^ suggesting that the MLI may serve as a reservoir for *Crassvirales*. In this study, we extended these finding by detecting *Crassvirales* in 40/51 participants, with *Crassvirales* viral reads representing up to 82.4% of an individual’s virome (47.5% by normalized relative abundance). While we were unable to identify statistically significant correlations between bacterial taxa and *Crassvirales*, suggesting that these may be either weak, context-dependent, or obscured by other factors influencing microbial community dynamics, our data does reveal that, in a pediatric (primarily adolescent) population, IBD is associated with a reduced relative abundance of *Crassvirales*. This observation is consistent with findings from studies in adult populations that reported decreased *Crassvirales* in individuals with CD^58^ as well as individuals with UC who did not respond to treatment.^20^ Interestingly, this shift was not identified with geNomad annotations alone and was only apparent after using a more robust effort to classify *Crassvirales* contigs using homology to conserved *Crassvirales* proteins; highlighting the ongoing importance in annotation strategies in virome research. However, we did not observe a significant correlation between *Crassvirales* abundance and local inflammation or specific IBD subtype.

**Figure 8.**
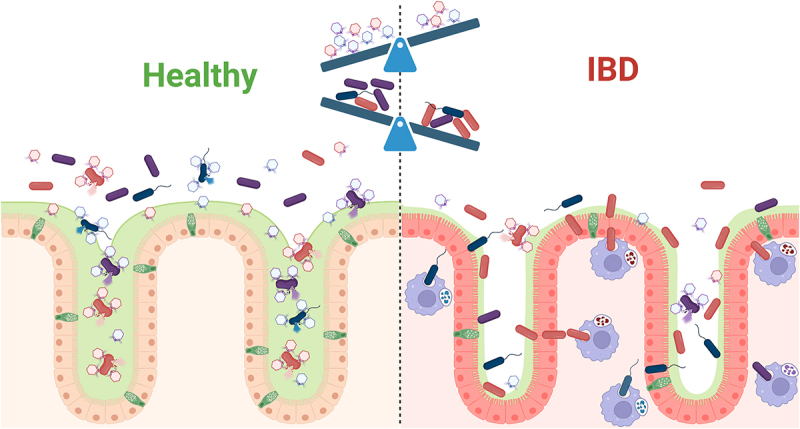
Proposed model of *Crassvirales* and its disease associations in IBD.

Collectively, our findings along with those from other studies suggest that *Crassvirales* may be markers of a healthy virome, and could potentially serve as biomarkers or modulators of virome health, independent of the local inflammatory status. The presence of *Crassvirales* and potentially other phages in the mucus layer may constitute an antimicrobial barrier that limits mucosal bacteria and protects the underlying epithelium. In addition, the mucus layer may dictate bacterial-virome relationships by allowing both microbes and viruses to co-exist due to physical separation (positive correlations) that could switch to predatory relationships (negative correlations) if depleted. In support of this hypothesis, we have previously reported on the exclusive presence of specific *Crassvirales* phage species within the MLI^27^ and bacteriophages adhering to the mucus have been shown to provide a non-host-derived immunity that actively protects the mucosal surface.^66^ Consistent with the essential role of the mucosal virome in host protection, mice humanized with non-IBD colon mucosal viromes were shown to be protected from intestinal inflammation.^26^ Our proposed model is summarized Figure 8.

In a healthy colonic mucosal-luminal interface, a thick mucus layer embeds *Crassvirales* and other phages, enabling their interaction with potentially pathogenic and proteolytic bacteria (red) that attempt to penetrate toward the epithelial layer. This interaction allows phages to initiate their lytic replication cycle, limiting the proximity of these bacteria to the mucosal surface and providing a protective barrier. Phages embedded within the mucus layer are also physically separated from beneficial microbes (purple), allowing both to stably coexist at high levels. In contrast, the thinner mucus layer observed in IBD results in reduced *Crassvirales* abundance within the mucosal layer. The reduction of *Crassvirales* and other phages permits the expansion of pathobionts, such as *Phocaeicola vulgatus*, which can directly interact with mucosal cells. The proteolytic enzymes produced by these pathobionts would contribute to increased epithelial damage, reduced intestinal barrier integrity, and enhanced bacterial translocation across the epithelial layer, thereby contributing to the inflammation characteristic of IBD. Thinner mucus layers would also result in more microbe-phage interactions and lead to predatory-prey interactions that may deplete beneficial microbes.

Based on these studies, a decrease in mucosal *Crassvirales* abundance could weaken the antimicrobial barrier, allowing the invasion of the mucus layer by *Crassvirales* bacterial hosts. *Crassvirales* are associated with bacteria from the phylum Bacteroidetes and, based on limited isolated virions, they appear to specifically infect *Bacteroides/Phocaeicola* genera.^67^ Interestingly, several *Bacteroides/Phocaeicola* species have been shown to induce colitis in mouse models susceptible to IBD.^68^ Furthermore, a recent study demonstrated that *P. vulgatus* and *P. dorei* can disrupt colonic epithelial integrity, leading to colitis, a phenotype associated with their proteases.^69^ Our observed increase in *P. vulgatus* in both IBD subtypes would support this hypothesis; although we note that several ASVs belonging to the *Bacteroides/Phocaeicola* genus, such as *P. dorei*, were not increased in CD/UC in this study and we were unable to identify specific *Crassvirales*/bacterial correlations. This could be due to differences in the specific species that we were able to identify using our 16S amplicon approach and/or due to sub-strain variation as both *P. vulgatus* and *P. dorei* have been reported to have extensive pangenomes (especially *P. vulgatus*) that could impact the infectivity of specific *Crassvirales* phages.^70^ Indeed, *P. vulgatus* genomes typically carry fewer CRISPR-Cas anti-phage systems and have a higher proportion of bacteriophages/insertions sequences as compared to *P. dorei*.^70^ It is thus tempting to speculate that the expansion of *P. vulgatus*, but not *P. dorei*, in IBD may be due to decreased selective pressure from endogenous *Crassvirales* phages. In addition, we found evidence for sex-specific differences in *P. vulgatus* abundances only in those diagnosed with CD or UC. This suggests that there are potentially sex-specific differences in the *Crassvirales* phage repertoires in pediatric IBD, as has been reported in studies on healthy individuals.^71^ Although we did not identify statistically significant differences in our dataset, these potential sex-associated variations could contribute to the high degree of virome personalization observed in this study. These findings highlight the potential significance of *Crassvirales* in protecting their host from colitis and underscore the need for further research to elucidate their role in maintaining virome health and mucosal immunity.

### Exploratory analysis of the intestinal virome’s longitudinal stability in IBD

While the human fecal virome has been reported to be individualized and longitudinally stable,^19,72,73^ those studies primarily involved healthy volunteers. In contrast, the gut virome is likely to experience greater temporal variation in the context of human illnesses, as suggested in fecal virome studies in individuals with IBD as well as those infected with COVID-19.^21,74^ Our study demonstrates that the mucosal virome remains individualized, even when comparing treatment-naïve microbiomes to samples taken several months later after initiation of IBD therapies. While most VCs were only present at diagnosis, the 2.4–9.3% of VCs which persisted were more likely to be abundant, often representing a majority of the viral community at repeat colonoscopy. Furthermore, the proportion of shared viruses appear to stabilize over the course of a year, suggesting the presence of both a subset of low-abundance, transient viruses and a persistent virome that resides in the colonic mucosa and remains stable over time, even as individuals transition from active disease with an inflamed mucosa to clinical remission with normal mucosa. The transient virome identified in this study may primarily result from the impact of disease treatment and reduced intestinal inflammation. However, it is unclear whether the long-term persistent virome identified in individuals with IBD has beneficial or detrimental consequences. This persistent virome could either represent the baseline virome expected in healthy individuals or a reservoir of phages that could promote future disease flares. Further research is necessary to elucidate the role of this persistent virome in IBD pathogenesis and its potential implications for disease management.

### Limitations and next steps

Our protocol is designed to identify DNA bacteriophages, which represents the majority of intestinal viruses. Bacteriophages are the primary drivers of virome diversity patterns in the gut, as opposed to eukaryotic viruses, which play a more limited role in shaping these dynamics.^59^ We thus did not identify enveloped viruses (which includes many eukaryotic viruses) or RNA viruses. It is important to note that our previous metatranscriptomics study demonstrated the absence of RNA-associated viral signatures in our samples.^27^ However, alterations in the RNA virome have been reported in IBD, particularly in individuals with Crohn’s disease experiencing clinical flares.^17^ Additionally, specific eukaryotic viruses, such as *Orthohepadnavirus*, have been associated with UC.^75^ Alternate sequencing or sampling approaches are required to comprehensively profile RNA viruses. Future studies employing these approaches will be critical to unraveling the contributions of RNA viruses and eukaryotic viruses to intestinal disease processes.

Our study also utilizes multiple displacement amplification (MDA) which introduces biases into both virome composition and diversity analyses. To address this issue, we have previously demonstrated the reliable quantification of a control spike-in phage using MDA.^27^ This control allows us to calibrate our amplification process and assess the accuracy of our virome measurements despite the inherent biases introduced by MDA. Nonetheless, the limitations of MDA remain a concern, particularly when analyzing samples with low microbial loads, such as those obtained from the gastrointestinal mucosa including sites proximal to the colon.^21,76^ While we were able to identify bacteriome-virome correlations, it is unclear whether these are direct (viral host interactions), indirect through other mediators or spurious correlations due to unknown factors. In addition, the virome at the MLI exhibited greater inter-individual variability than the bacteriome, indicating that larger cohort sizes may be necessary to detect statistically significant correlations as compared to typical bacteriome studies. The collection and use of MLI samples are a strength of this study. The mucosal layer of the gastrointestinal tract represents a critical niche for understanding the gut virome, particularly in the context of IBD where the mucosa is a site of active inflammation and altered microbial composition. Unlike the luminal environment, the mucosa provides a unique microenvironment where bacteriophages constitute an antimicrobial barrier,^66^ and microbial communities, including viruses, engage directly with the epithelial barrier and immune system. Previous studies have revealed that several viral members of the gut microbiome, including *Crassvirales*, possess mucus-interacting capabilities, enabling them to attach to mucin.^77^ Despite these observations, the mechanisms underlying these interactions and their potential effects on host physiology and disease processes remain largely unexplored. Sampling the mucosal layer offers the unique opportunity to investigate these critical virome-microbes dynamics. However, sampling from the bowel following the clean-out required for safe colonoscopy cannot fully represent native conditions.^78^ Nevertheless, it allowed us to investigate the inner mucus composition regionally and assess the tightly adherent MLI communities.^38^ The higher-resolution sampling of MLI samples adds a further layer of heterogeneity with further potential impact of varying local disease severity and different spatial patterns of inflammation. However, due to the invasive nature of endoscopic sampling, we lack a traditional “healthy cohort” commonly used in fecal virome studies. Instead, our non-IBD controls may include individuals with underlying conditions that could introduce potential confounding factors, even in the absence of mucosal inflammation.

This study provides valuable insights that can guide future studies involving larger, standardized clinical cohorts that include matched proximal and distal colon, as well as fecal samples. Such an approach will be instrumental in advancing microbiome studies beyond solely utilizing fecal specimens, addressing a critical gap in viromics research. In particular, understanding the exact role of the virome at the site of IBD disease remains a key challenge, as specific phages may exert either beneficial or pathogenic effects. Moreover, the extent to which the virome shapes, or is influenced by, disease states such as active inflammation, remission, and flares is still poorly understood. Future work using large cohorts of participants with IBD with repeated samplings over time will likely be required to answer these questions and overcome the high interpersonal variability seen within the colonic virome.

## Supplementary Material

supplementary_table_03.xlsx

supplementary_table_01.xlsx

supplementary_figure_01.jpg

supplementary_table_02.docx

supplementary_table_04.xlsx

supplementary_figure_02.png

## Data Availability

Host-removed, high-quality sequencing reads are available under BioProject PRJNA1004560.

## Abbreviations

CD: Crohn’s disease
DC: distal colon
IBD: inflammatory bowel disease
MLI: mucosal-luminal interface
NRA: normalized relative abundance
PC: proximal colon
PCDAI: Pediatric Crohn’s Disease Activity Index
PUCAI: Pediatric Ulcerative Colitis Activity Index
SES-CD: Simplified Endoscopic Activity Score for Crohn’s disease
UC: ulcerative colitis
wPCDAI: weighted Pediatric Crohn’s Disease Activity Index

## Resource availability

Lead contact: Further information and requests for resources and reagents should be directed to and will be fulfilled by the lead contact, Alain Stintzi (astintzi@uottawa.ca).

Materials availability: This study did not generate new unique reagents.

Date and code availability: The datasets generated during this study are available at NCBI PRJNA1004560.
