# Supplementary figures and images for: The colonic mucosal virome in inflammatory bowel disease reveals Crassvirales depletion and disease-specific virome features
Source: Gut Microbes. 2025 Aug 3;17(1):2539450. doi: 10.1080/19490976.2025.2539450 (PMC12323425; doi:10.1080/19490976.2025.2539450)

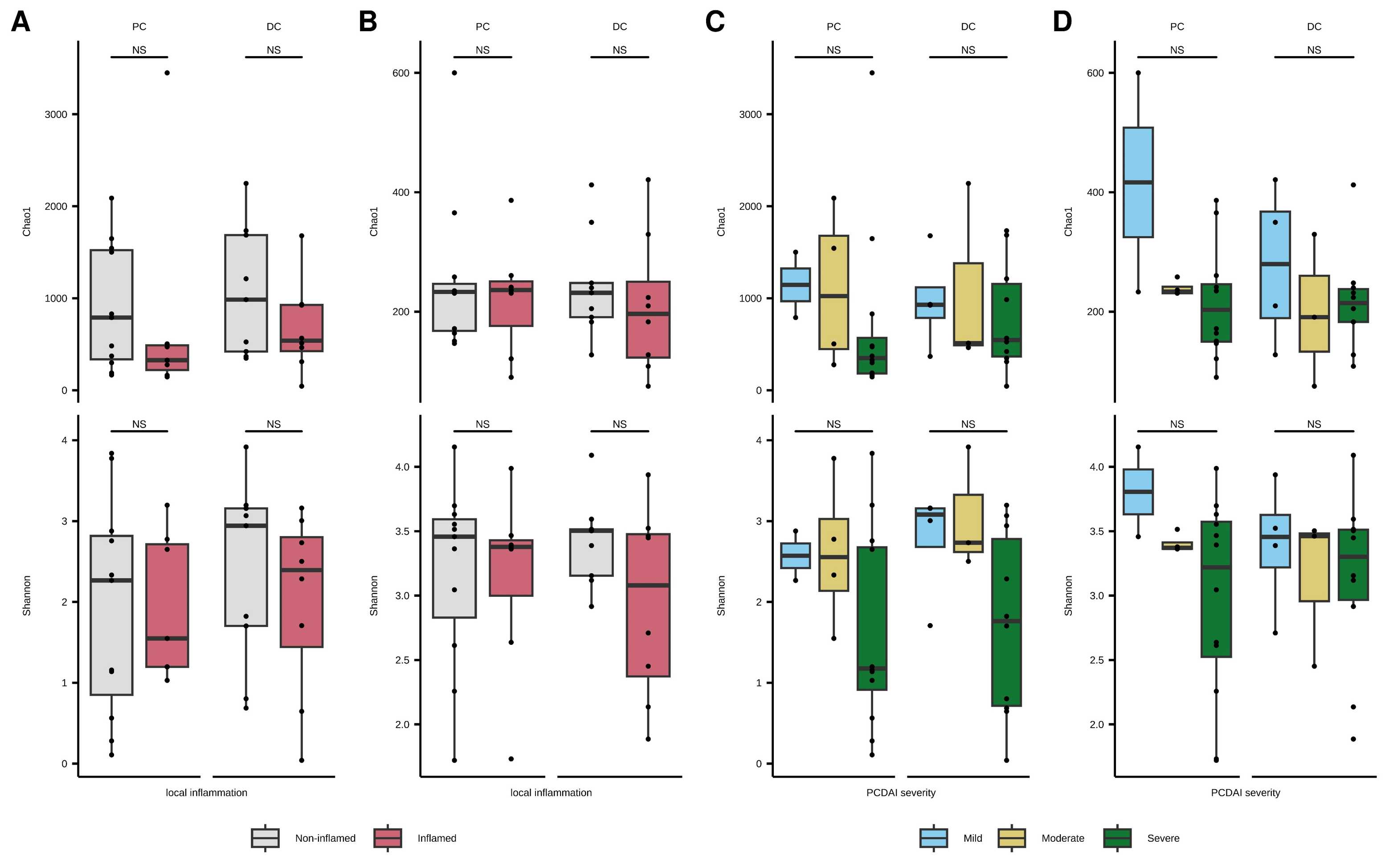

Supplement: supplementary_figure_01.jpg [file KGMI_A_2539450_SM2733.jpg]

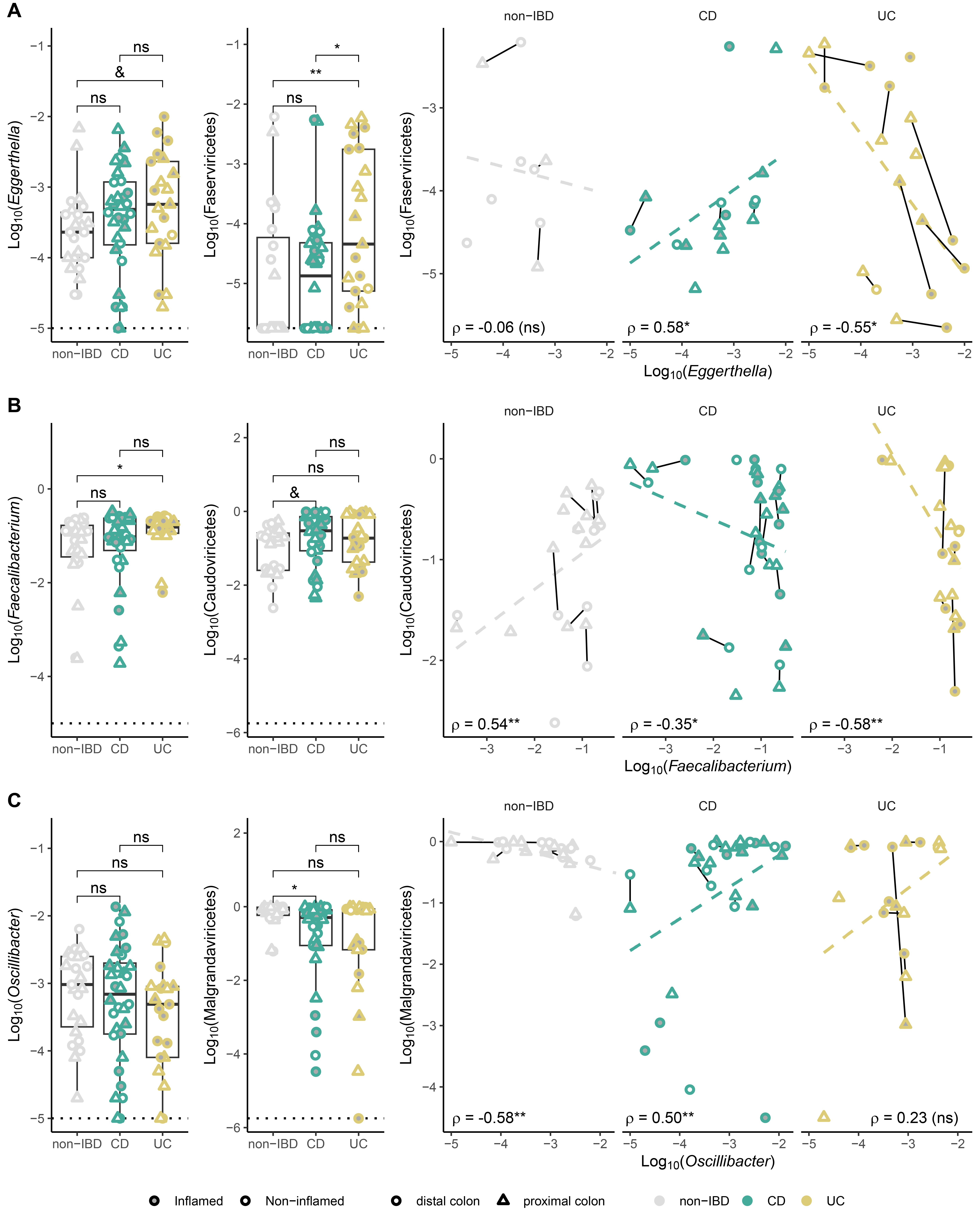

Supplement: supplementary_figure_02.png [file KGMI_A_2539450_SM2730.png]
