# Supplementary material for: The colonic mucosal virome in inflammatory bowel disease reveals Crassvirales depletion and disease-specific virome features
Source: Gut Microbes. 2025 Aug 3;17(1):2539450. doi: 10.1080/19490976.2025.2539450 (PMC12323425; doi:10.1080/19490976.2025.2539450)
Supplement: supplementary_table_02.docx [file KGMI_A_2539450_SM2732.docx]

**Supplementary Table 2: Differential abundance analysis of viral taxa (viral class and viral order/family) using geNomad annotations, comparing non-IBD, CD, and UC participants.** The taxonomy structure is based on geNomad 1.5.0, where viral class is reflected by the fifth taxonomic rank, while the sixth taxonomic rank includes a mix of viral orders and families (without viral order), as listed in the table below. fdr: false-discovery rate adjustment. Values under 0.05 are bolded, with the categories contributing to viral taxa enrichment/depletion described underneath. Viral taxa present in fewer than 3 samples were removed from this analysis

|  | proximal colon | | | | distal colon | | | |
| --- | --- | --- | --- | --- | --- | --- | --- | --- |
| **Viral class** | By diagnosis (Kruskal-Wallis) | | By inflammation  (Wilcoxon) | | By diagnosis (Kruskal-Wallis) | | By inflammation  (Wilcoxon) | |
|  | **p_value** | **fdr** | **p_value** | **fdr** | **p_value** | **fdr** | **p_value** | **fdr** |
| Arfiviricetes | 0.2738 | 0.3834 | 0.6235 | 0.6491 | 0.4391 | 0.4815 | 0.9629 | 0.9629 |
| Caudoviricetes | 0.1456 | 0.2549 | 0.4794 | 0.6491 | **0.0343**  🡫 in non-IBD | 0.0800 | 0.1925 | 0.4931 |
| Faserviricetes | 0.0555 | 0.1639 | 0.5651 | 0.6491 | 0.4815 | 0.4815 | 0.1754 | 0.4931 |
| Malgrandaviricetes | **0.0483**  🡩 in non-IBD | 0.1639 | 0.4341 | 0.6491 | **0.0319**  🡩 in non-IBD | 0.0800 | 0.2113 | 0.4931 |
| Megaviricetes | 0.3535 | 0.4124 | 0.6491 | 0.6491 | 0.4682 | 0.4815 | 0.3640 | 0.6370 |
| Repensiviricetes | 0.5167 | 0.5167 | 0.3692 | 0.6491 | 0.3201 | 0.4815 | 0.8593 | 0.9629 |
| unannotated | 0.0703 | 0.1639 | 0.5271 | 0.6491 | **0.0297**  🡫 in non-IBD | 0.0800 | 0.6469 | 0.9056 |

|  | proximal colon | | | | distal colon | | | |
| --- | --- | --- | --- | --- | --- | --- | --- | --- |
| **Viral order/family** | By diagnosis (Kruskal-Wallis) | | By inflammation  (Wilcoxon) | | By diagnosis (Kruskal-Wallis) | | By inflammation  (Wilcoxon) | |
|  | **p_value** | **fdr** | **p_value** | **fdr** | **p_value** | **fdr** | **p_value** | **fdr** |
| Ackermannviridae | 0.0691 | 0.2108 | 0.3692 | 0.8178 | 0.2122 | 0.4548 | 0.2548 | 0.6371 |
| Algavirales | 0.7067 | 0.7572 | 0.8327 | 0.8922 | 0.9048 | 0.9503 | 0.8375 | 0.9629 |
| Arfiviricetes_no-order | 0.5167 | 0.7046 | 0.3692 | 0.8178 | 0.2122 | 0.4548 | 0.2548 | 0.6371 |
| Autographiviridae | 0.7815 | 0.7815 | 0.8967 | 0.8967 | 0.7042 | 0.8802 | 0.8593 | 0.9629 |
| Caudoviricetes_no-order | 0.1435 | 0.3588 | 0.7569 | 0.8733 | 0.1869 | 0.4548 | 0.4711 | 0.9629 |
| Cirlivirales | 0.2687 | 0.5038 | 0.5997 | 0.8178 | 0.6152 | 0.8389 | 0.9629 | 0.9629 |
| Crassvirales | 0.6572 | 0.7572 | 0.7389 | 0.8733 | 0.9430 | 0.9503 | 0.9459 | 0.9629 |
| Geplafuvirales | 0.5167 | 0.7046 | 0.3692 | 0.8178 | 0.5360 | 0.8041 | 0.8593 | 0.9629 |
| Imitervirales | 0.0691 | 0.2108 | 0.4507 | 0.8178 | 0.1975 | 0.4548 | **0.0375**  🡩 with inflammation | 0.5624 |
| Mulpavirales | 0.5167 | 0.7046 | 0.3692 | 0.8178 | 0.5360 | 0.8041 | 0.8593 | 0.9629 |
| Petitvirales | **0.0483**  🡩 in non-IBD | 0.2108 | 0.4341 | 0.8178 | 0.0672 | 0.4548 | 0.2113 | 0.6371 |
| Rountreeviridae | 0.5721 | 0.7151 | 0.4507 | 0.8178 | 0.9503 | 0.9503 | 0.7472 | 0.9629 |
| Straboviridae | 0.1928 | 0.4131 | 0.4087 | 0.8178 | 0.4937 | 0.8041 | 0.2548 | 0.6371 |
| Tubulavirales | 0.0555 | 0.2108 | 0.5651 | 0.8178 | 0.2005 | 0.4548 | 0.1754 | 0.6371 |
| unannotated | 0.0703 | 0.2108 | 0.5271 | 0.8178 | 0.0817 | 0.4548 | 0.6468 | 0.9629 |
